# Supporting ColoREctal Equitable Navigation (SCREEN): a protocol for a stepped-wedge cluster randomized trial for patient navigation in primary care

**DOI:** 10.1186/s43058-024-00598-5

**Published:** 2024-06-03

**Authors:** Jessica N. Rivera Rivera, Katarina E. AuBuchon, Laura C. Schubel, Claire Starling, Jennifer Tran, Marjorie Locke, Melanie Grady, Mihriye Mete, H. Joseph Blumenthal, Jessica E. Galarraga, Hannah Arem

**Affiliations:** 1 Healthcare Delivery Research Network, MedStar Health Research Institute, Washington, DC USA; 2 Lombardi Comprehensive Cancer Center, Georgetown University, Washington, DC USA; 3 Department of Medicine, MedStar Washington Hospital Center, Washington, DC USA; 4 Department of Surgery, MedStar Washington Hospital Center, Washington, DC USA; 5 MedStar Health Institute for Quality and Safety, Washington, DC USA; 6 Department of Behavioral Health Research, MedStar Health Research Institute, Washington, DC USA; 7 Department of Psychiatry, Georgetown University School of Medicine, Washington, DC USA; 8 Center for Biostatistics, Informatics and Data Science, MedStar Health Research Institute, Washington, DC USA; 9 Department of Health Equity, MedStar Health, Columbia, MD USA; 10 Department of Oncology, Georgetown University, Washington, DC USA

**Keywords:** Colorectal cancer screening, Primary care, Stepped-wedge design, Patient navigation, Colorectal cancer disparities

## Abstract

**Background:**

Black individuals in the United States (US) have a higher incidence of and mortality from colorectal cancer (CRC) compared to other racial groups, and CRC is the second leading cause of death among Hispanic/Latino populations in the US. Patient navigation is an evidence-based approach to narrow inequities in cancer screening among Black and Hispanic/Latino patients. Despite this, limited healthcare systems have implemented patient navigation for screening at scale.

**Methods:**

We are conducting a stepped-wedge cluster randomized trial of 15 primary care clinics with six steps of six-month duration to scale a patient navigation program to improve screening rates among Black and Hispanic/Latino patients. After six months of baseline data collection with no intervention we will randomize clinics, whereby three clinics will join the intervention arm every six months until all clinics cross over to intervention. During the intervention roll out we will conduct training and education for clinics, change infrastructure in the electronic health record, create stakeholder relationships, assess readiness, and deliver iterative feedback. Framed by the Practical, Robust Implementation Sustainment Model (PRISM) we will focus on effectiveness, reach, provider adoption, and implementation. We will document adaptations to both the patient navigation intervention and to implementation strategies. To address health equity, we will engage multilevel stakeholder voices through interviews and a community advisory board to plan, deliver, adapt, measure, and disseminate study progress. Provider-level feedback will include updates on disparities in screening orders and completions.

**Discussion:**

Primary care clinics are poised to close disparity gaps in CRC screening completion but may lack an understanding of the magnitude of these gaps and how to address them. We aim to understand how to tailor a patient navigation program for CRC screening to patients and providers across diverse clinics with wide variation in baseline screening rates, payor mix, proximity to specialty care, and patient volume. Findings from this study will inform other primary care practices and health systems on effective and sustainable strategies to deliver patient navigation for CRC screening among racial and ethnic minorities.

**Trial registration:**

NCT06401174

**Supplementary Information:**

The online version contains supplementary material available at 10.1186/s43058-024-00598-5.

Contributions to the literature:

- Research shows that patient navigation programs can reduce cancer screening disparities, but the scale of such programs is limited.
- Our study builds on the NCI Cancer Moonshot’s Accelerating Colorectal Cancer (CRC) Screening and follow-up through Implementation Science (ACCSIS) eight studies to provide additional context for scaling CRC navigation specifically focusing on Black and Hispanic/Latino populations across a diverse urban healthcare system.
- Our protocol specifies exact training protocols and data collection systems separately for intervention modifications and implementation strategies to capture the full scope of any changes to inform future programs.

## Background

The United States Preventative Service Task Force (USPSTF) recommends that all people aged 45–75 years old receive colorectal cancer (CRC) screening. Common methods to complete screening include stool-based tests such as those that look for blood and DNA (every 3 years), blood only (annually), or colonoscopy, a procedure performed in a clinical setting (every 10 years if normal screening or as follow up to a positive stool sample test) [1]. Despite convincing evidence that CRC screening can prevent CRC mortality by preventing or detecting cancer in early stages, there are challenges to achieving the 2030 healthy people goal to increase screening to 68.3% of the population compared to the 2021 rate of 58.7% [2].

In addition to increasing overall screening rates, to advance health equity there is an urgent need to address the disparities in screening rates and navigation to follow up care by race and ethnicity. In the United States (US) CRC is the second leading cause of cancer death for Hispanic/Latino people, [3, 4] yet in 2021, only 50.8% of Hispanic/Latino screening-eligible adults reported up-to-date CRC screening relative to 60.9% and 61.1% for non-Hispanic White and Black people, respectively [5]. Hispanic/Latino individuals often cite cultural deterrents from screening including stigma, fear, embarrassment, and a perception that only males need screening [6–8]. Hispanic/Latino immigrants in particular may be faced with lack of services in their native language, reduced access to employer-provided health insurance, or limited time off from work, ultimately leading to reduced healthcare access [9]. Despite higher screening rates than Hispanic/Latino individuals, Black individuals in the US are at increased risk of being diagnosed with CRC, [3] and have increased mortality from CRC compared to other racial and/or ethnic groups [10]. A recent simulation study demonstrated that CRC incidence differences between Black and White populations are driven by screening disparities [11]. Experiences of discrimination [12] and low trust due to historical medical racism are often cited by Black individuals as a barriers to timely CRC screening and any necessary follow-up care [13, 14].

Previous research demonstrates that patient navigation narrows inequities across the cancer care continuum, [15] including gaps in screening among Black and Hispanic/Latino patients [16–23]. Still, despite strong evidence that patient navigation improves CRC screening uptake, there is limited implementation of patient navigation, even within integrated healthcare systems. Primary care clinics are an ideal venue to deliver patient navigation to advance equitable screening rates given that there is ongoing patient contact which may increase trust as well as opportunities for closing the loop on screening completion, as well as provider and healthcare system-based incentives linked to quality metrics. Clinics considering patient navigation for CRC screening may need to design programs differently by clinic characteristics (patient demographics or payors, clinic location, electronic health record capabilities, volume of providers and staff turnover or training programs). Thus, tailored implementation strategies may be needed to enable delivery across different primary care clinics to advance equitable delivery.

### Current study

This study's primary objective is to determine the effectiveness of the CRC screening patient navigation program for navigating Black and Hispanic/Latino patients in 15 primary care clinics using a stepped-wedge cluster randomized clinical trial. The study was named “**S**upporting **C**olo**RE**ctal **E**quitable **N**avigation”, or SCREEN. Given the established effectiveness of patient navigation for increasing CRC screening and a desire to measure implementation strategies across multiple settings, we did not employ a parallel cluster randomized trial, but rather selected a stepped wedge design so that all clinics eventually receive the intervention. We will simultaneously describe implementation strategies and protocol adaptations across 15 participating primary care sites. We hypothesize that our patient navigation program will increase CRC screening rates and decrease CRC screening disparities among Black and Hispanic/Latino individuals receiving primary care. We also anticipate that the intervention will show high acceptability, feasibility, and appropriateness from multilevel stakeholders, but that clinics will have different preferences for implementation strategies. The study was registered in clinicaltrials.gov (NCT06401174) on May 3rd, 2024.

## Methods

### Formative work

The proposed intervention stems from a current hospital-based primary care navigation program within the integrated healthcare system that is based on the New Hampshire Colorectal Cancer Screening Program (NHCRCSP) [24]. As part of our study to adapt and scale this program with a particular focus on advancing health equity, we engaged in formative work to ensure a contextual and culturally-responsive design. We thus conducted interviews with healthcare system physicians and advance practice practitioners, as well as operational leaders and clinic managers, to identify perceived barriers and opportunities to support patient navigation workflows across diverse settings. We also interviewed Black and Hispanic/Latino patients who were eligible for CRC screening to understand knowledge, concerns, barriers, facilitators, and recommendations regarding CRC screening. Interview findings are beyond the scope of the protocol paper and will be reported elsewhere. In addition, we assembled a Community Advisory Board (CAB) for this study composed of five community-based organizations and advocacy groups, and separately created an advisory board of patient representatives who are between the ages of 45–75 and self-identify as Black or Hispanic/Latino. The advisory boards will meet monthly for the first three months to provide feedback on the study design, navigation protocol, progress, and to aid in setting priorities and centering the needs of Black and Hispanic/Latino people in the DC area and then transition to quarterly meetings all together with interim check-ins with stakeholders as needed.

### Conceptual frameworks

**Fig. 1:**
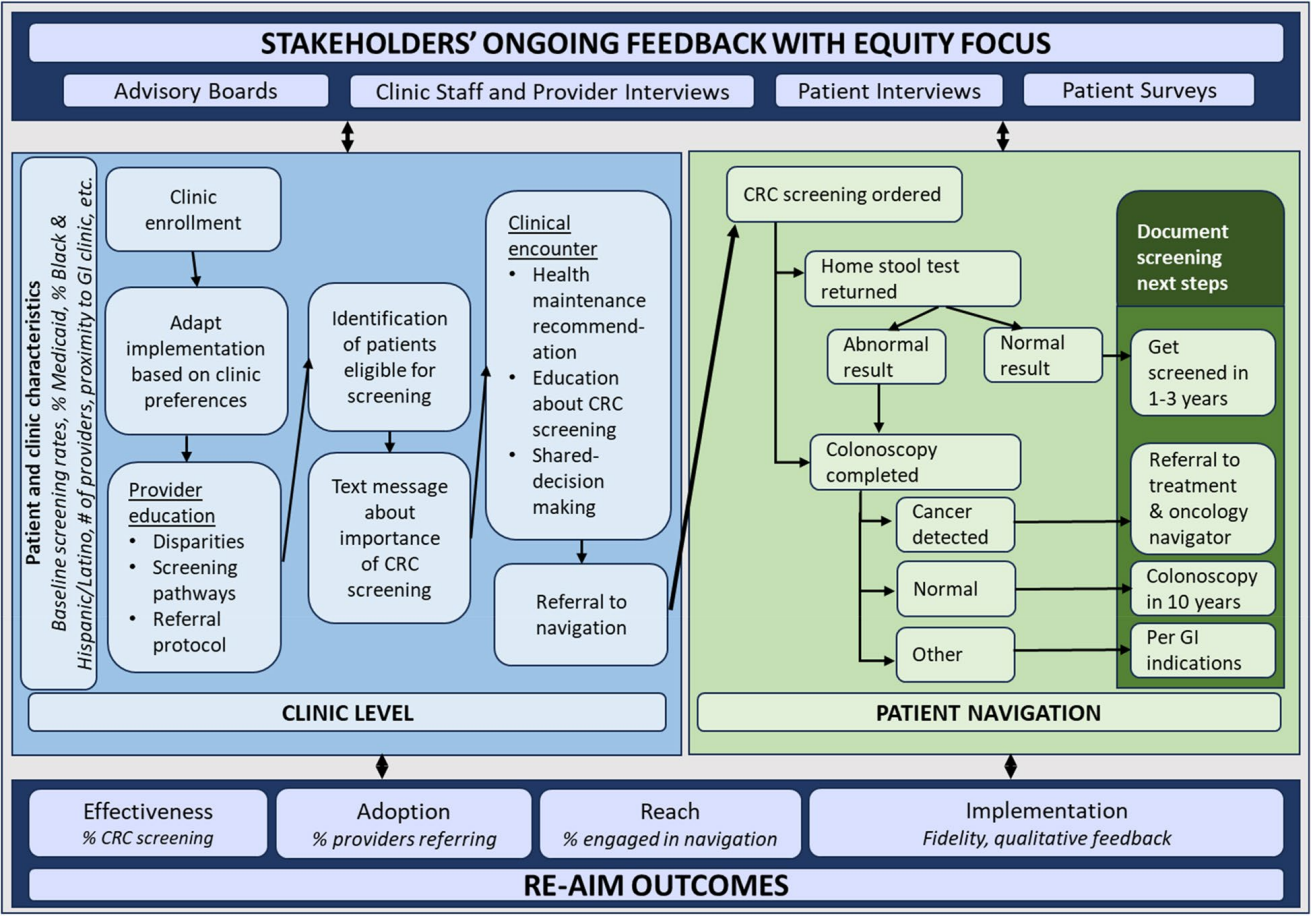
PRISM-based Conceptual Framework for SCREEN

This study’s conceptual framework is centered on the Practical, Robust Implementation and Sustainment Model (PRISM) [25] and applies the Cancer Prevention and Control Research Network’s (CPCRN) nine health and racial equity principles [26, 27]. To improve CRC screening rates among Black and Hispanic/Latino patients, we will involve stakeholders, clinics and patients in the development, adaptation, and evaluation of the CRC screening patient navigation intervention (Fig. 1). Based on the PRISM components we will evaluate the intervention effectiveness (comparing the number and percentage screened in the intervention vs control conditions), adoption (number of providers referring to navigation), reach (number of patients referred out of those who are eligible overall and by intersectional patient characteristics such as age, insurance, and language preference), and implementation (adaptations made during study delivery, fidelity, qualitative feedback on the program). We will also describe the implementations strategies employed to understand expanded opportunities for scaling this evidence-based CRC screening patient navigation intervention across diverse settings. We apply the nine CPCRN health and racial equity principles (Table 1) through engagement with our Advisory Board (capacity building, understanding community priorities, establishing transparent relationships) and with a system level approach to investigating and addressing disparities (exploring systems and root causes of cancer disparities, prioritizing sustainability of research benefits, center racial equity, engage in equitable data collection, analyses, interpretation, and dissemination) and partnership with the National Committee on Quality Assurance (NCQA) to assess and suggest best practices for community engagement in this type of work.

**Table 1:** Application of health and racial equity principles in the Cancer Prevention and Control Research Network

Principle	Study Delivery
Engage in power-sharing and capacity building with partners	Discussions with community organizations and grassroots leaders in application development around existing programs, gaps, and community engagement
Address community priorities through community engagement and co-creation of research	In application development, identification of how the proposal could support partners in achieving their health equity objectives, and ongoing input on navigation approach through the community advisory board
Explore and address the systems and structural root causes of cancer disparities	Formative work in first six months of project including interviews to better understand multi-stakeholder perspectives on systems and structural causes of disparities in CRC screening
Build a system of accountability between research and community partners	Commitment to existing partners (e.g. Federally Qualified Health Centers) to utilize health system resources to navigate patients who need follow up care in the healthcare system and to optimize data sharing
Establish transparent relationships with community partners	We have formal contracts with our community partners documenting responsibilities, expectations, and benefits to participation, we will promote community partner engagement in dissemination
Prioritize the sustainability of research benefits for community partners	Engaging health system leadership and National Committee for Quality Assurance (NCQA) to focus on sustainability including capacity for navigation referrals from community partners and will work closely with our community advisory board on how to benefit community partners
Center racial equity in cancer prevention and control research	Study designed to include Black and Hispanic/Latino individuals, historically marginalized populations that have worse screening rates and/or CRC outcomes than their white counterparts
Engage in equitable data collection, analysis, interpretation, and dissemination practices	NCQA will help guide the analytic plan, synthesis, and dissemination of results; team will conduct reflexive participant collaboration to interpret results with advisory board
Integrate knowledge translation, implementation, and dissemination into research plan	Distribute results through traditional channels such as conferences and peer review publications, as well as through community events and direct to patients; we will send ongoing project updates out to stakeholders; we will present to the Health Equity Leaders Coordinating Council which includes executive sponsors and champions for health equity within the healthcare system

#### Clinic eligibility and recruitment

During study planning we met with primary care leadership within the healthcare system to ensure buy in for the proposed approach. To select primary care clinics for participation, we reviewed baseline screening rates overall and by race/ethnicity, patient volume, geographic location, and overall clinic percentage of patients with Medicaid. Given significant diversity in these characteristics across the healthcare system we sought to engage clinics that either had a lower than median overall screening rate, or an identified disparity in screening by key factors such as race (compared to White), ethnicity (Hispanic/Latino) or payor (compared to private insurance). We purposively selected a list of 20 primary care clinics to oversample our primary population of Black and Hispanic/Latino patients due for CRC screening and engaged with primary care leadership to confirm the appropriateness of our proposed list and to ensure buy in. We then conducted outreach by email, including engaging our clinical co-investigators to reach out to the 20 clinic leads and offering to meet with clinic representatives to discuss the study until a total of fifteen clinics agreed to participate. Only one clinic declined participation due to staffing turnover. We stopped pursuing the recruitment emails once we reached our target of fifteen clinics. Included clinics are listed on the clinicaltrials.gov website page.

#### Cluster-randomized controlled trial

**Fig. 2:**
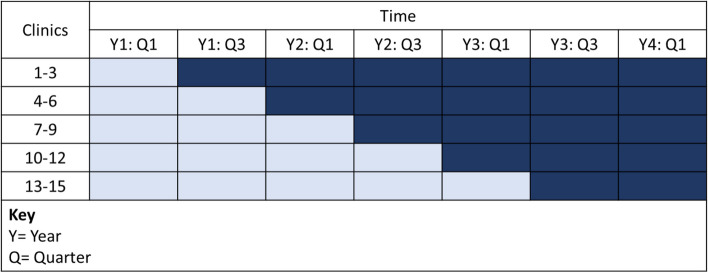
Stepped-wedge study design: Schedule of events for clusters to start the intervention

The statistician (MM) will randomize all clinics stratified by clinic size (categorized as small, medium, large number of Black and Hispanic/Latino patients due for screening) into five, six-month steps (three clinics per step) (Fig. 2). In the stepped wedge design, after a six month baseline period where all clinics are in the control arm, clinics will cross-over to the intervention condition in sequential, staggered fashion with three clinics per step until all clinics have received the intervention. In each step, clinics randomized to intervention will receive focused implementation support for six months to tailor the patient navigation program. After the six-month step, clinic patients will continue to receive navigation and providers will continue to receive reminders and communication, but feedback will move to a quarterly cadence.

#### Intervention arm core clinic components

Overarching strategies are outlined in Table 2. Once the clinic is assigned to the intervention arm, they will be asked to designate two clinic champions per clinic including one physician or advanced practice provider (APP) and one person who would manage daily workflows such as a practice manager or senior medical assistant. These individuals will complete a survey on organizational readiness to change [28]. Champions will also answer basic questions about workflow related to CRC screening. Within the first month of rolling out the program at a new clinic the patient navigators and study team members will schedule a site visit to review project goals including cultural tailoring and relevance for Black and Hispanic/Latino populations, provide provider and patient facing materials about the navigation program, and complete any baseline clinic level assessments that may affect how the navigation program is delivered. Providers will be asked to refer Black and Hispanic/Latino patients age 45 to 75 years old who have previously missed screening or require assistance to complete screening due to transportation, stigma, health literacy, or other barriers.

**Table 2:** Example implementation strategies

Strategy category	Individual strategy	Study application
Evaluative and iterative	Assess for readiness and identify barriers and facilitators	Clinic level assessments upon joining the intervention arm
	Audit and provide feedback	Utilize internal dashboard to provide regular updates on CRC screening by race/ethnicity to providers within intervention clinics
Develop stakeholder relationships	Build a coalition	Work with the community advisory board to understand root causes of disparities, review proposed intervention materials, ongoing review to best serve community members
Engage consumers	Intervene with patients to enhance uptake and adherence	Conduct formative interviews with patients and collect ongoing feedback on experiences with intervention; outreach by phone and patient portal
Change infrastructure	Change record systems	Utilize OMOP Common Data Model to define outcome; create referral orders specific to individual primary care sites
Train and educate stakeholders	Conduct educational meetings with stakeholders	Meet with clinical champions including physicians and administrators to review project goals and solicit feedback; engage system leadership

During the six-month active support period, the study team will review weekly data and will reach out to clinics if two sequential weeks of continuous data during the six-month of active support period show that providers are not referring eligible patients to navigation in order to meet the minimum sample size of 20 patients per clinic per six months. We will meet with the clinical champions first and if needed will conduct additional site visits to troubleshoot, develop additional supportive materials, or consider how communication or reminders through the electronic health record (EHR) might be needed to support referrals to patient navigation. Conversely, it is possible that we will receive too many referrals, whereby it appears that providers are referring everyone due for screening rather than those who have a history of not completing screening or identified barriers to screening. If this is the case, we will consider providing decision support tools for triaging who needs referral or creating a system within our team to stratify intensity of support based on the number of previous referrals/orders that were incomplete or identification of social needs in the medical record.

The research team will review data bi-weekly and communicate with the clinical champions at least monthly during the initial six months in the intervention arm. This data will include progress of CRC patient navigation referrals and completion of screening. After the six-month intensive support, they will receive quarterly updates on CRC screening completion rates overall and by provider, broken down by patient race/ethnicity.

#### Intervention arm optional clinic components

Additional clinic level implementation strategies may take different forms for each clinic in terms of ancillary supports or frequency of feedback and iterative refinement. These optional strategies including additional approaches to engaging patients, clinic assessments and tailoring, audit and feedback loops. Examples of optional supports include customized tracking of referrals or orders, appointments, and test results, tailoring patient or provider communication strategies, additional site visits, educational materials, and rapid reviews of specific patient panels and process metrics for quality improvement. The optional arm components will be closely tracked by the research study team.

### Patient navigation- Patient-level component

#### Patient eligibility and initial CRC outreach

Patients will be eligible if they are 45 to 75 years old, identify as Hispanic/Latino and/or Black, are not up to date on CRC screening recommendations, and have had at least one primary care visit at the included clinics in the past 365 days. The target population of patients who are due for screening is intended to align with the quality metrics relevant to CRC screening in the healthcare system. While we are not limiting to annual well visits, we will conduct sensitivity analyses to understand how reason for a visit affects screening referral, navigation referral, or completion outcomes. For clinics in the intervention phase, the provider will have the option to refer patients to navigation. Two days prior to their primary care appointment, patients will receive a reminder via text message indicating that their CRC screening is due and encouraging them to talk to their doctor about CRC screening.

#### Patient navigation strategy

For colonoscopy we adapted a patient navigation program our team (ML & JT) conducts at a single hospital-based primary care clinic which utilizes the evidence-based NHCRCSP. The home-based stool tests navigation strategy was informed by relevant NHCRCSP strategies and materials, the Cologuard navigation program offered by the manufacturer Exact Sciences, and our team’s current patient navigation program (Table 3).

**Table 3:** Patient navigation schedule by test type

Colonoscopy	Stool Test (FIT or Cologuard)
Topic 1. Engagement, CRC Screening Education and Barrier Assessment (3–5 days of navigation assignment) *Navigator follows up about patient’s consult appointment*	Topic 1. Engagement, CRC Screening Education and Barrier Assessment (within 5 days of navigation assignment)
Topic 2. Prep Education and Barrier Resolution (2 weeks prior procedure)	Topic 2: Kit Confirmation and Barrier Resolution (1 week after ordering kit)
Topic 3. Prep Review and Re-Addressing Barriers (5–7 days prior procedure)	Topic 3. Re-addressing Barriers (if results are not back after 2 weeks of ordering kit)
Topic 4. Assessment of Prep and Confirmation of Test Day Details (2–3 days prior to procedure)	Topic 4. Follow-up about results and next steps *Patients with positive test results are navigated for colonoscopy*
Topic 5. Follow-up, patient understanding of results and next steps (1–2 weeks after procedure)	

We reviewed existing literature, [29] community and national organizations’ resources, [30–39], obtained feedback from our CAB, formative stakeholder interviews, and research team to identify relevant culturally sensitive materials and tailor our patient navigation materials and implementation strategies. We will maintain an emphasis on patient decision-making about the different types of CRC screening (where appropriate) and how to address cancer stigma when talking about CRC screening. Patient navigators will assess social and practical barriers affecting completion of CRC screening including language, health literacy, medical transportation, or financial instability. To address patients’ barriers for completing CRC screening, we will create a list of available community resources (e.g. transportation) for the clinics assigned to the intervention arm to assist in CRC screening completion.

All navigators will be trained and supervised by a registered nurse with expertise in patient navigation for CRC screening (ML). Informed by the NHCRCSP Patient Navigation Training, [40] the navigators will complete training for CRC screening during the first four weeks of hire (Table 4).

**Table 4:** Patient navigator training

Areas	Activities
Research	• CITI trainings (includes HIPAA and confidentiality)
Computer, phone, and documents	• EHR access and documentation training • Navigation monitoring (REDCap) access and training • Scheduling access and training • Endoscopy software access and training for different GI clinics • Cell phone use and voice mail • Language line training
CRC screening	• Review *Screen for Life* materials • Review CDC colorectal cancer website • Full review of USPSTF guidelines • Comprehensive overview of CRC screening and surveillance, including details of screening test options and patient risk assessment • Review Fight Colorectal Cancer CRC screening pre-recorded webinars (Prep recommendations, CRC screening, and CRC screening among African American and Medically Underserved Communities) • Basic pathology overview by an experienced nurse navigator • FIT training by PCP provider • Cologuard training by Exact Sciences
Navigation	• Review available supportive services (e.g., translation, transportation) • Shadow experienced navigators • Motivational interviewing training • Cultural Humility & Language Access Training • Learn about tricks for successful stool-test sample and bowel prep • Enroll and participate in DC Primary Care Association for peer group navigation training webinars • Physician Data Query (PDQ) Cancer Information for Health Professionals • National Colorectal Cancer Roundtable Messaging Guidebooks
Clinics	• Observe consult and colonoscopy at a GI clinic
Supervision	• Navigator trainee will begin making patient calls with experienced navigator observing to provide feedback • Ongoing feedback to trainees via weekly group supervision

#### Patient feedback

At the end of the navigation, for quality improvement purposes, patients will be asked to complete a Patient Satisfaction Survey with Likert scale responses for overall satisfaction, specific program components, and likelihood of recommending the program to friends and family.

### Measures and outcomes

#### Primary outcome

Our primary effectiveness outcome is clinic-level CRC screening completion rates every six-months for Black and Hispanic/Latino patients aged 45 to 75 years old comparing intervention and control conditions (within and across clinics). Our grant partner, the National Committee on Quality Assurance (NCQA) will lead translation of the HEDIS® colorectal cancer screening (COL) quality measure from the Fast Healthcare Interoperability Resources data model into the Observational Medical Outcomes Partnership (OMOP) common data model, supporting efficient, reliable, outcome measurement to support translation of findings into policy-aligned practice. This will include development of all existing COL cohort definition in ATLAS, [41] creation of the ATLAS concepts (using the HEDIS Value Set Directory as a primary reference, without external publication or replication of those value sets), and creation of the COL artifacts for implementation in the MedStar Health dataset. Definitions and concepts will undergo iterative review with both NCQA and MedStar Health teams to ensure accuracy and implementation feasibility. These Observational Health Data Sciences and Informatics query definitions can be run at any institution and will be made publicly accessible. All data on completion of CRC screening by race and ethnicity will be extracted from the EHR under a HIPAA waiver. We will also consider intersectional characteristics of participants such as age and payor in addition to race and ethnicity. We will compare EHR data extracted manually to follow quality metric standards with data obtained through the OMOP Common Data Model to cross-validate results.

#### Secondary outcomes

We will also assess implementation outcomes including reach and adoption of the culturally tailored navigation intervention. Reach will be assessed as the percentage of eligible Black and Hispanic patients at each clinic who received a referral for navigation, and of those referred, who engaged with navigation. While we will look at overall reach, we will also calculate the number of people referred among those who have previously failed to complete screening referrals/orders to better understand navigation referral for those who are at higher risk of non-compliance. We will consider intersectional factors affecting reach such as patient insurance, age, race/ethnicity, and primary language. Adoption will be assessed by the percentage of providers who referred eligible patients for navigation. Each of these outcomes will also be examined separately for Black and Hispanic/Latino patients.

We will solicit feedback from clinical champions both on patient navigation and the implementation strategies (engaging patients, clinic assessments and tailoring, audit and feedback loops) via annual surveys. Surveys will include questions on acceptability, feasibility, and appropriateness using a validated 12-item scale, [42] the Clinical Sustainability Assessment Tool, [43] questions on satisfaction with the SCREEN programs, and suggestions for improvement. At the end of the trial, we also will conduct interviews with one or two representatives from each clinic site to understand how the intervention and implementation strategies addressed or failed to address contextual barriers to navigation implementation.

To track changes to the implementation strategies, or clinic-level delivery of navigation, we will adopt the Longitudinal Implementation Strategy Tracking System (LISTS, Table 4) [44], which incorporates implementation science strategy reporting and specification standards, [45] and the framework for documenting modifications to implementation strategies (FRAME-IS), [46] to understand how an intervention is being delivered and iteratively adapted across diverse settings. These methods will support the documentation of the implementation strategies so that we can better understand implementation of a patient navigation program for historically underserved populations across clinics with different baseline characteristics.

##### Navigation fidelity

Navigators will track phone calls in REDCap [47]. The REDCap form will include a structured list of key components that should be covered by type of phone call (Supplemental Table 1), and the navigator will select each of the key components that were covered in the phone call. We will also conduct fidelity checks of navigation bi-annually by a research team member observing a randomly selected 5% of phone calls with a structured checklist to record fidelity to the prescribed intervention.

##### Patient-level

We will conduct online surveys in years 2 and 4 across all 15 clinics to understand patient experiences of care using the Discrimination in Medical Setting Scale [48] via REDCap. At Year 2, we will survey 20 patients per clinic (*n* = 300) who have not undergone navigation and in Year 4 we will survey 20 patients per clinic (*n* = 300) who completed navigation. In Year 4, we will also include a survey about acceptability of the navigation and open-ended questions about the navigation experience and appropriateness of cultural tailoring. During the intervention phase in Years 2 to 4, we will interview 10–15 patients a year who complete navigation, to understand experiences in navigation and to get feedback on the intervention.

### Data analysis plan

Baseline characteristics of the patients in the intervention and control groups will be summarized first in a traditional way (intervention vs control) using means, standard deviations for continuous variables and frequencies and percentages for categorical variables. Exploratory descriptive analyses will also be conducted using clinic level data for six-month periods to examine patient characteristics to ensure a balanced design in an intent-to-treat (ITT) framework. Initial comparisons between the intervention and control groups will be done using two-sample test statistics such as t-test and Chi-square as appropriate. The effectiveness of the intervention will be assessed based on the primary patient-level outcome, screening completion, which is measured as a binary variable indicating whether a patient identified as “due for screening” completed CRC screening, and will be analyzed using Generalized Linear Latent and Mixed Models [49] suitable for categorical, ordered or count outcomes with random effects at multiple levels since observations will be nested in clinics and clinics will be exposed to intervention at different time points. The model will include a treatment indicator, a fixed effect for time (steps) and their interactions to account for the potentially confounding effect of varying crossover timepoints for clinics. The model will also include patient characteristics (Black or Hispanic/Latino, age, sex, payor) as well as random effects for clinics. Secondary patient and clinic level outcomes will be described and compared between intervention and control groups using the methods described earlier. We will also include Area Deprivation Index in the models as appropriate, calculated based on 2020 census data. Analyses will be conducted in Stata or R. All analyses will consider sex as a biological variable and race as a social construct.

The study was powered to detect a significant difference in the CRC screening rates between Black and Hispanic/Latino patients who receive navigation services and those who do not receive navigation. The design consists of 15 clusters and 6 steps (one baseline all clusters in control condition + 5 intervention steps) of six-month duration where 3 clusters will switch to intervention every 6-month. A sample size of an average of 120 participants (60 intervention and 60 control) per cluster and an average of 20 participants per cluster/step will achieve 80% power with 1800 participants (900 navigation and 900 control participants) to detect a conservative 10-percentage point difference in the primary outcome measure of CRC screening completion. The navigation intervention is expected to increase the screening rate from an average 60% (current data across clinics) to an average of 70%. The test statistic used was the two-sided Wald Z-test, the intra-class correlation (ICC within-clinic) was assumed to be 0.05 and the Type I error was 0.05. Sample size calculations were conducted in PASS 15 [50]. Since the navigation will be applied to all referrals and we will obtain screening completion data from EHR, this sample size will be considered as the minimum number of patients to achieve the anticipated effect size.

## Discussion

The described patient navigation CRC screening protocol is intended to address the disparities in CRC screening uptake among the Black and Hispanic/Latino population. While patient navigation is known to improve CRC screening among Black and Hispanic/Latino people, [51] more research is needed to understand the implementation strategies needed to promote uptake of navigation and active CRC screening outreach. By using longitudinal implementation strategy tracking, this project we will provide unique and insightful data on adaptation to scaling patient navigation for CRC screening across 15 diverse primary care clinics, and will help elucidate how this CRC screening navigation program can scale to additional settings or preventative programs.

Our study comes at a time of both overall healthcare system and national interest in increasing CRC screening rates. Within our healthcare system there is growing attention to identifying and addressing disparities in access to care, including CRC screening as a metric of interest. At the national level, as part of the Cancer Moonshot, the National Cancer Institute has funded an initiative called Accelerating CRC Screening and Follow up through Implementation Science including eight research projects across the nation to target implementation of multi-level evidence-based programs [52]. These secular changes and emerging findings will be monitored by the study team and any emerging programs or trends will be accounted for via study process documentation and where needed post-hoc in analyses looking at changes over time.

Patient navigation programs have shown to be one of the most effective strategies to improve CRC screening uptake among the Black and Hispanic/Latino population, in addition to interventions that address structural barriers such as transportation or employment/scheduling concerns by providing stool-based kits [16, 22, 53]. Comprehensive activities through the core navigation intervention include patient CRC screening education and reminders, assessing and providing resources for CRC screening barriers, supporting and motivating patients, scheduling appointments for patients, and guiding patients through screening completion. As part of this intervention, we will also facilitate stool-based kits in-clinic and by mail, use culturally sensitive CRC screening education materials (videos and flyers), and receive ongoing feedback from our advisory boards. Centered on patient navigation, this intervention also incorporates different clinic protocols to optimize CRC screening outcomes [54].

This protocol is poised to make a significant contribution to the literature on implementation of evidence-based approaches using multilevel strategies to scale navigation delivery with both rigorous randomized design and health equity focused solicitation of stakeholder input. Still, we anticipate some possible challenges during the study. If we do not receive the expected number of clinic referrals, we will work with the clinics to outline who should be referred to the navigation program (e.g. using EHR-generated lists to identify failure to complete stool-based screening within 60 days). If a clinic decides to stop their participation in this intervention prior to intervention step, we will document this and move forward without replacing the clinic. Following the ITT principle, we will still obtain screening data for that clinic from the EHR and use the randomization assignment for that clinic as planned. There are also established limitations to EHR data, including challenges in identifying orders for stool tests and documented completion of results. We used the baseline period to verify EHR data extraction code with quality control review by clinical experts. We also are working with NCQA on developing OMOP code to verify results. We anticipate that through ongoing quality control we will be able to describe and remedy most of the EHR challenges in defining outcomes.

In the final project year NCQA will co-lead synthesis of study results, focusing on the translation of findings to national quality improvement efforts. Specifically, NCQA will evaluate the study’s community engagement and intervention approach and identify how this approach can scale to broader national implementation through existing policy and payment mechanisms. Grant-funded community-based research efforts are frequently critiqued for their high susceptibility to funding cycles, with efforts stalling when research funding runs out. To ensure sustainability, efforts must be integrated into programs, systems, and payment models for care. First, NCQA will work with members of the CAB to identify key criteria of success from the community perspective for engaged intervention design. Second, NCQA will evaluate existing national quality programs (measures, standards) at the health system and health plan levels, identifying how elements of the intervention strategy align or differ from these standards. Third, NCQA will review the study tracking logs including implementation strategies to inform how navigation delivery worked in real world settings. Finally, NCQA will assess and describe strategies, in this policy environment, for health care systems to effectively resource and implement community-informed methods for CRC screening that prioritize success criteria as identified by community partners.

## Conclusion

To advance health equity we must design studies to target disparities in colorectal cancer screening and promote fairness and social justice. We anticipate that results of this project will have implications within our healthcare system for sustaining funding for patient navigation programs and that lessons learned through process evaluation will inform other primary care systems looking to implement similar programs.

### Supplementary Information

Supplementary Material 1.

## Data Availability

Any specific training materials will be made available upon request. There is no data available at this time.

## Abbreviations

APP: Advance practice practitioners
CAB: Community Advisory Board
COL HEDIS®: Colorectal cancer screening
CPCRN: Cancer Prevention and Control Research Network’s
CRC: Colorectal cancer
EHR: Electronic health record
FHIR: Fast Healthcare Interoperability Resources
FRAME-IS: Framework for documenting modifications to implementation strategies
ITT: Intent-to-treat
LISTS: Longitudinal Implementation Strategy Tracking System
NCQA: National Committee for Quality Assurance
NHCRCSP: New Hampshire Colorectal Cancer Screening Program
OMOP: Observational Medical Outcomes Partnership
PRISM: Practical, Robust Implementation and Sustainment Model
US: United States
USPSTF: United States Preventative Service Task Force
